# USING GEOGRAPHIC INFORMATION SYSTEMS ANALYSIS TO UNDERSTAND THE BURDEN OF PROVIDING RIDES FOR OLDER ADULTS

**DOI:** 10.1093/geroni/igae098.3924

**Published:** 2024-12-31

**Authors:** Rebecca Mauldin, Swasati Handique, Soeun Jang, Rupal Parekh

**Affiliations:** University of Texas at Arlington, Arlington, Texas, United States; University of Texas at Arlington, Arlington, Texas, United States; University of Texas at Arlington, Arlington, Texas, United States; University of Connecticut, New York, Connecticut, United States

## Abstract

Transportation is a significant factor for older adults’ mobility and quality of life. However, some older adults need rides from others for transportation and ride providers may experience burdens when providing rides regularly. This study explores the use of geographic information systems to develop geospatial models (or “activity spaces”) of routine travel patterns, both for regular activities and for providing transportation to older adults. This exploratory study was a cross-sectional survey among people who provided rides to older Vietnamese immigrants in the Dallas-Fort Worth metroplex area (N=20). Geospatial analyses using ArcGIS were employed to examine activity spaces (AS) and the geospatial burden of providing rides. Results indicated that nearly half of the ride providers experienced transportation costs, missed work, and increased personal stress from giving rides. Significant portions of ride-provision activity spaces were found outside the provider’s regular activity spaces. We present and compare three potential indicators for assessing ride providing burden based on the ride providers’ regular and ride-provision activity spaces. The percentage of the ride-provision AS outside the AS was associated with giving fewer rides per month. Findings suggest potential ways to measure ride provider burden for research and practice and have the potential to inform transportation subsidy policies. This study has implications a policy subsidizing private ride providers which could reduce costs and improve accessibility without additional infrastructure. Geospatial tools can assess ride-provision burdens, guide subsidy eligibility, and provide valuable data for city planning and service location optimization.

